# Association of Suicide Risk With Headache Frequency Among Migraine Patients With and Without Aura

**DOI:** 10.3389/fneur.2019.00228

**Published:** 2019-03-19

**Authors:** Yu-Kai Lin, Chih-Sung Liang, Jiunn-Tay Lee, Meei-Shyuan Lee, Hsuan-Te Chu, Chia-Lin Tsai, Guan-Yu Lin, Tsung-Han Ho, Fu-Chi Yang

**Affiliations:** ^1^Department of Neurology, National Defense Medical Center, Tri-Service General Hospital, Taipei, Taiwan; ^2^Beitou Branch, Department of Psychiatry, National Defense Medical Center, Tri-Service General Hospital, Taipei, Taiwan; ^3^National Defense Medical Center, School of Public Health, Taipei, Taiwan

**Keywords:** migraine, suicide, suicidal ideation, suicide attempts, depression

## Abstract

**Background:** Migraines with aura have been associated with suicide in adolescents and young adults, but the association between suicide and migraine frequency has not been determined. This study investigated suicidal ideation and suicide attempts among patients with varying frequencies of migraines, with and without auras.

**Methods:** This cross-sectional study analyzed 528 patients aged between 20 and 60 years from a headache outpatient clinic in Taiwan. All patients completed a set of questionnaires, including a demographic questionnaire, the Migraine Disability Assessment questionnaire, the Hospital Anxiety and Depression Scale, the Beck Depression Inventory, and the Pittsburgh Sleep Quality Index. Suicide risk was evaluated by self-reported lifetime suicidal ideation and attempts. Patients were divided into low-frequency (1–4 days/month), moderate-frequency (5–8 days/month), high-frequency (9–14 days/month), and chronic (≥15 days/month) migraine groups. The association between migraine frequency and suicidality was investigated using multivariable linear regression and logistic regression.

**Results:** The rates of suicidal ideation and suicide attempts were the highest for chronic migraine with aura (ideation: 47.2%; attempts: 13.9%) and lowest in migraine-free controls (2.8%). Migraine frequency was an independent risk factor for suicidal ideation and attempts in patients with aura (both *P*_trend_ < 0.001), but not in patients without auras. Migraine aura and depression were associated with higher risks of suicidal ideation and suicide attempts in patients with migraine.

**Conclusion:** High migraine frequency has a correlation with high suicide risk in patients who experience an aura, but not in other patients with migraine.

## Introduction

Migraine is the second most common primary headache, affecting approximately 19.1% of women and 9% of men in the United States (1). The disorder is characterized by recurrent attacks of moderate-to-severe throbbing or pulsatile sensations on one side of the head, lasting 4–72 h. The characteristic features of migraine are sensitivity to light (photophobia) and sound (phonophobia), nausea and vomiting, and aggravation by physical activity (2). According to the Global Burden of Disease Study, migraine is the third leading cause of disability in people aged under 50 years and the first among neurological disorders (3, 4). Migraine is comorbid with several psychiatric conditions, including depression, anxiety, and sleep disorders (5, 6). Studies have suggested an association between migraine and suicide (7–9); however, the relationship between migraine frequency and suicide risk remains uncertain.

Suicide is a serious but at least partially preventable public health problem. According to the Global Burden of Disease Study, approximately 842,000 people worldwide commit suicide annually. In the United States, self-harm is the seventh leading cause of years of life lost (10). Coexisting psychiatric disorders such as depression, anxiety, and sleep disorders are strong predictors of suicide (11–13). In addition, employment status, alcohol use, and marital status have been linked to suicide (14, 15). Migraine is often comorbid with depression, anxiety, and sleep disorders, and thus suicide risk may be a critical problem in patients with migraine.

Previous studies have established a relationship between suicide and migraine (9, 16). Breslau et al. showed that in a cohort of young adults aged 21–30 years, those with migraine and auras had elevated rates of suicide attempts and suicidal ideation (9). In a community-based study of Taiwanese adolescents aged 13–15 years, Wang et al. found that migraine with aura was associated with suicidal ideation (16). However, these studies have failed to consider other migraine characteristics and confounding factors that affect suicide risk such as anxiety, depression, and sleep quality.

In the present study, we hypothesized that suicidal ideation and suicide attempts are associated with migraine frequency, regardless of the presence of aura. We investigated relationships between suicide risk and migraine characteristics while controlling for comorbid psychiatric diseases, alcohol use, educational level, and marital status.

## Methods

### Patients

This cross-sectional study included 528 consecutive patients aged between 20 and 60 years attending a headache clinic at the Department of Neurology of the Tri-Service General Hospital (TSGH) in Taipei, Taiwan, between June 2015 and May 2017. All participants signed a written informed consent form after a full written and verbal explanation of the study. The study protocol was approved by the Institutional Review Board of the TSGH. Patients with migraine, both with and without aura, were analyzed. After exclusion of patients with concomitant primary or secondary headaches, those for whom the amount of data to determine migraine frequency was insufficient, and those with missing information regarding clinical characteristics, 528 patients were included in the final analysis. Of these, 96 patients experienced chronic migraine (≥15 days/month), 122 had high-frequency migraine (9–14 days/month), 106 had medium-frequency migraine (5–8 days/month), and 204 had low-frequency migraine (1–4 days/month). Of the 528 patients with migraine, 170 (32.2%) had migraines with aura and the remaining 358 had migraines without aura. In addition, 177 healthy volunteers who fulfilled the following criteria were selected as a control group: no family history of migraine and no previous diagnosis of other primary or secondary headache disorders, except for infrequent episodic tension-type headaches (<6 days/year). Patients who completed a screening questionnaire were subsequently interviewed by a board-certified neurologist and headache specialist to make a diagnosis according to the International Classification of Headache Disorders, 3rd edition (ICHD-3 beta) (2). Patients with migraine were determined to be with or without aura, based on the criteria of the International Headache Society (2).

A previous study reported proportions of suicide attempts of 9.1 in patients with migraine and 2.6 in healthy controls (7). Given the alpha level of 0.05, power of 0.80, and allocation ratio of 3, the obtained minimum sample size was 444 and 148 individuals in the migraine and control groups, respectively. Sample size calculation was performed using Power and Sample Size version 08 (NCSS, LLC, Kaysville, UT, USA).

### Questionnaires

After obtaining written informed consent, the patients were interviewed; they then completed a structured questionnaire packet with regard to demographic data, including age, sex, body mass index (BMI), education level, marital status, employment status, and alcohol consumption. They also completed the Migraine Disability Assessment questionnaire (MIDAS) (17), the Hospital Anxiety and Depression Subscales (HADS) (18), the Beck Depression Inventory (BDI) (19), and the Pittsburgh Sleep Quality Index (PSQI) (20). This study used the Chinese versions of the MIDAS, HADS, BDI, and PSQI, all of which had been validated as having high sensitivity and specificity (21–24). The MIDAS score is based on a 5-item questionnaire and assesses headache-related disability over the previous 3 months (17). The HADS is a 14-item scale used to measure anxiety (7 items) and depression (7 items). Each item is coded from 0 to 3 (0, not at all; 1, sometimes; 2, often; and 3, all the time), providing a maximum subscale score of 21 for anxiety and depression. The BDI is a 21-question multiple-choice survey with a maximum score of 63. The PSQI assesses sleep quality over the most recent 1-month interval. It consists of 19 individual items and 7 component scores, summed to produce a final score. Suicidal ideation and suicide attempts were assessed by asking the following questions used by Posner et al. (25, 26): Suicidal ideation—*Have you had any thoughts about killing yourself?* Suicide attempts—*Did you ever do anything to try to kill yourself or do anything else that you knew might end your life?*

### Data Analysis

We present data for continuous variables as mean ± standard deviation and for categorical variables as frequency and proportion. Differences in categorical variables across the study groups (control, 1–4 days, 5–8 days, 9–14 days, and ≥15 days) were tested using the Cochran–Armitage chi-square test. Linear trends in the distribution of variables across the study groups were tested using the general linear model for continuous variables. To evaluate the associations between migraine frequency and the prevalence of suicidal ideation and suicide attempts, both a univariate analysis (the Cochran–Armitage chi-square test) and a linear contrast test in the multivariable logistic regression analysis with adjustment of subject characteristics (the variables listed in Table 1 were fully adjusted except for aura and MIDAS) were conducted. The multicollinearity among the predictors was checked using the variance inflation factor which a value >10 was considered to have serious problem of collinearity.

**Table 1:** Characteristics of the study population.

		**Episodic migraine**				
**Variable**	**Control**	**1–4 days/month**	**5–8 days/month**	**9–14 days/month**	**≥15 days/month**	***P* value^§^**
Sample size	177	204	106	122	96	–
Aura	–	63 (30.9)	36 (34.0)	35 (28.7)	36 (37.5)	0.484
Female sex	89 (50.3)	130 (63.7)	65 (61.3)	87 (71.3)	66 (68.8)	<0.001
Age (years)	35.4 ± 11.3	33.7 ± 10.3	34.2 ± 11.0	34.3 ± 11.0	32.7 ± 11.9	0.111
BMI (kg/m^2^)	23.7 ± 4.6	22.0 ± 3.5	22.6 ± 3.5	22.6 ± 3.7	22.4 ± 4.1	0.066
Education level (years)	14.7 ± 2.1	14.5 ± 2.8	15.1 ± 2.2	14.9 ± 2.2	14.2 ± 2.8	0.380
Marital status						0.513
Single	101 (57.1)	108 (52.9)	55 (51.9)	66 (54.1)	53 (55.2)	
Have a partner	71 (40.1)	85 (41.7)	49 (46.2)	51 (41.8)	36 (37.5)	
Divorced/widowed	5 (2.8)	11 (5.4)	2 (1.9)	5 (4.1)	7 (7.3)	
Employed status						0.801
Unemployed	5 (2.8)	6 (2.9)	6 (5.7)	11 (9.0)	4 (4.2)	
Employed	162 (91.5)	185 (90.7)	93 (87.7)	100 (82.0)	84 (87.5)	
Retired/homemaker	10 (5.6)	13 (6.4)	7 (6.6)	11 (9.0)	8 (8.3)	
Alcohol consumption						0.067
Never	109 (61.6)	130 (63.7)	56 (52.8)	86 (70.5)	66 (68.8)	
<Once a month	56 (31.6)	66 (32.4)	45 (42.5)	34 (27.9)	27 (28.1)	
≥1 day/week	12 (6.8)	8 (3.9)	5 (4.7)	2 (1.6)	3 (3.1)	
MIDAS	–	20.7 ± 18.1	30.1 ± 21.3	39.3 ± 28.5	72.8 ± 65.0	<0.001
HADS-anxiety	4.8 ± 2.8	7.4 ± 4.1	7.7 ± 4.1	8.2 ± 3.9	8.5 ± 4.4	<0.001
HADS-depression	3.5 ± 2.6	5.3 ± 3.7	5.1 ± 4.0	6.3 ± 3.8	6.8 ± 4.4	<0.001
BDI total score	4.2 ± 3.0	9.3 ± 7.3	9.9 ± 7.4	11.3 ± 7.9	12.4 ± 9.0	<0.001
PSQI total score	6.5 ± 2.9	8.2 ± 3.6	8.9 ± 3.6	9.5 ± 3.6	10.2 ± 4.0	<0.001

§, *Linear trend of Cochran–Armitage chi-square test for categorical variable or linear contrast in the general linear model for continuous variable*.

*MIDAS, Migraine Disability Assessment questionnaire; HADS, Hospital Anxiety and Depression Subscales; BDI, Beck Depression Inventory; PSQI, Pittsburgh Sleep Quality Index*.

Furthermore, a pre-specified subgroup analysis of the presence or absence of aura was performed. Finally, we performed both univariate and multivariable logistic regression analyses to identify potentially predictive factors of suicidal ideation and suicide attempts in patients with migraine. A two-sided *P*-value < 0.05 was considered statistically significant. No adjustment was made for multiple testing (multiplicity) in this study. The data analyses were performed using SPSS 22 (IBM SPSS, Armonk, NY, USA: IBM Corp.).

## Results

### Patient Characteristics

The study cohort comprised 177 subjects in the control group (25.1%) and 528 patients in the migraine groups (74.9%). Table 1 lists the characteristics of individuals in the control group and migraine groups. Among those with migraine, 170 patients experienced auras (32.2%) before the migraine episodes. The trend in the distribution of aura, age, BMI, education level, marital status, employment status, and alcohol consumption was non-linear across the study groups. The proportion of female patients was higher in all the study groups (50.3% in the control group and 68.8% in patients with chronic migraine; *P*_trend_ < 0.001). Univariate linear trend analyses also showed that higher migraine frequency had a correlation with higher MIDAS scores, HADS-anxiety subscores, HADS-depression subscores, BDI total scores, and PSQI total scores.

### Associations of Migraine Frequency With Suicidal Ideation and Suicide Attempts

**Figure 1:**
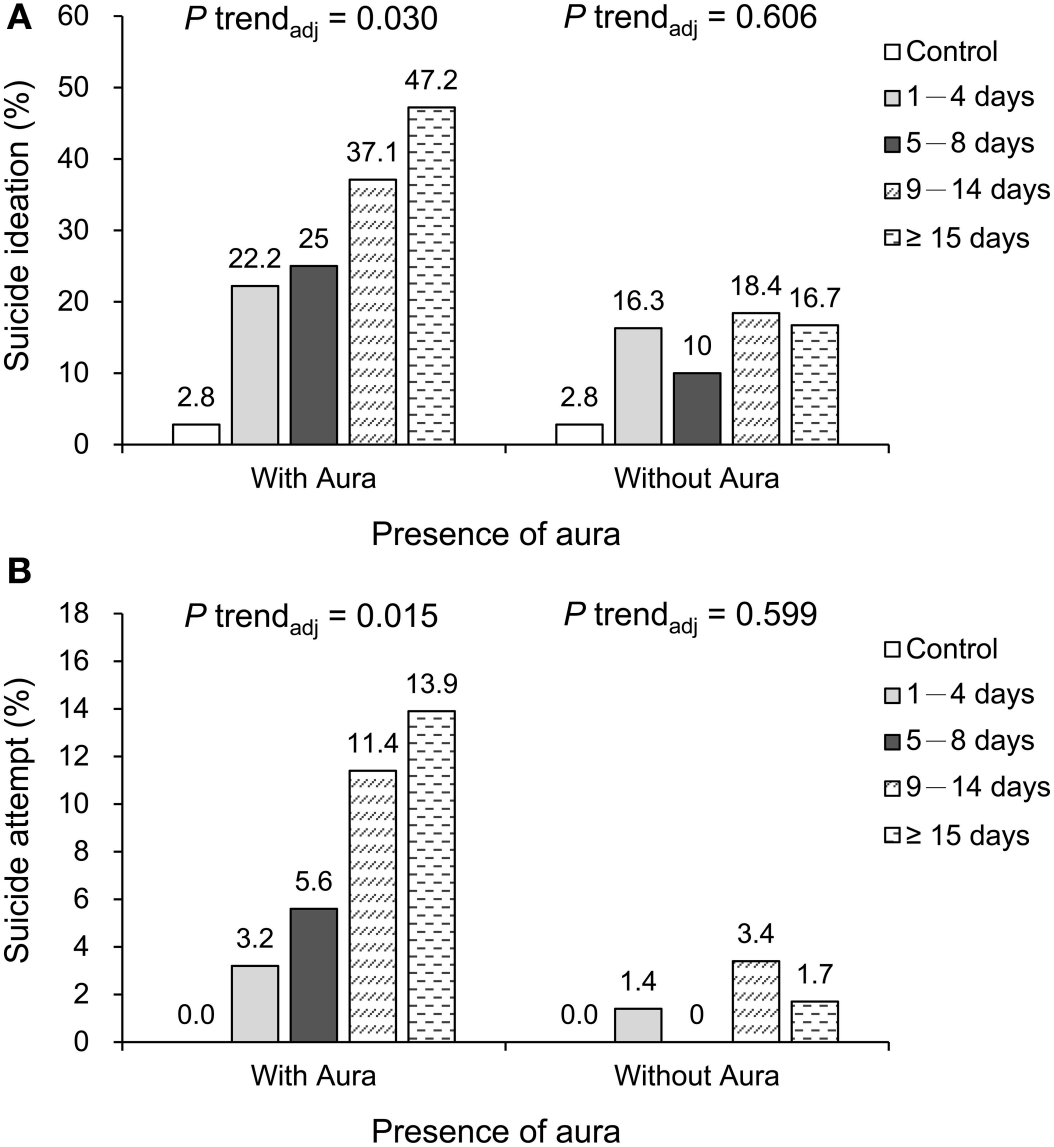
Prevalence of suicidal ideation **(A)** and attempted suicide **(B)** across the study groups, including patients with and without auras.

Five individuals in the control group (2.8%) and 109 in the migraine groups (20.6%) reported suicidal ideation, whereas 19 patients (3.6%) in the migraine groups said that they had attempted suicide. Table 2 summarizes the prevalence of suicidal ideation and suicide attempts across the study groups. The associations between migraine frequency and the prevalence of suicidal ideation were observed in the univariate analysis (*P* < 0.001), but in the multivariate analysis, these were non-significant, after adjusting for baseline characteristics (*P* = 0.137). Patients with migraine were further stratified into aura and non-aura groups, and the trend effect was tested again. The trend was significant across the study groups in patients with aura (*P*_multivariable_ = 0.030), but not in the groups without aura (*P*_multivariable_ > 0.05). The results for suicide attempts were similar to those for suicidal ideation. Figure 1A shows the prevalence of suicidal ideation (Figure 1A) and suicide attempts (Figure 1B) across the study groups, stratified according to presence of an aura.

**Table 2:** Prevalence of suicidal ideation and suicide attempts in the control and migraine groups.

		**Episodic migraine**					
**Outcome / subgroup**	**Control**	**1–4 days/month**	**5–8 days/month**	**9–14 days/month**	**≥15 days /month**	***P* value^§^**	***P* value^‡^**
**SUICIDAL IDEATION**							
Total	5 (2.8)	37 (18.1)	16 (15.1)	29 (23.8)	27 (28.1)	<0.001	0.137
With aura	0 (0.0)	14 (22.2)	9 (25.0)	13 (37.1)	17 (47.2)	<0.001	0.030
Without aura	5 (2.8)	23 (16.3)	7 (10.0)	16 (18.4)	10 (16.7)	<0.001	0.606
**SUICIDE ATTEMPT**							
Total	0 (0.0)	4 (2.0)	2 (1.9)	7 (5.7)	6 (6.3)	<0.001	0.103
With aura	0 (0.0)	2 (3.2)	2 (5.6)	4 (11.4)	5 (13.9)	<0.001	0.015
Without aura	0 (0.0)	2 (1.4)	0 (0.0)	3 (3.4)	1 (1.7)	0.064	0.599

§, *Linear trend of Cochran–Armitage chi-square test*;

‡, *Linear contrast in the logistic regression adjusted for age, sex, marital status, years of education, employment status, alcohol consumption, Hospital Anxiety, and Depression Subscales; Beck Depression Inventory scores, Hospital Anxiety and Depression Subscales for anxiety and depression, and Pittsburgh Sleep Quality Index total scores*.

In addition, we also provided the primary results according to gender in the Supplemental Tables 1, 2. The correlations of migraine frequency with suicidal ideation and suicide attempts were observed only in women with aura after adjustment of covariates (*P* for trend = 0.027 and 0.015 for suicidal ideation and suicide attempts, respectively). However, no correlation was observed in men with aura, possibly partially because of the predominance of women in the cohort (62%; 437/705).

### Factors Associated With Suicidal Ideation and Suicide Attempts

Table 3 lists the results of multivariable logistic regression analyses identifying the potentially predictive factors of suicidal ideation and suicide attempts in patients with migraine. The results showed that the presence of an aura and a higher BDI total score were independently predictive of suicidal ideation and that the presence of an aura, lower education level, and higher BDI total scores were independently predictive of suicide attempts.

**Table 3:** Multivariable logistic regression analysis of factors associated with suicidal ideation and suicide attempts in patients with migraine.

**Variable**	**Suicidal ideation**		**Suicide attempts**	
	**OR (95% CI)**	***P* value**	**OR (95% CI)**	***P* value**
**MIGRAINE FREQUENCY**				
1–4 days/month	Reference	–	Reference	–
5–8 days/month	0.72 (0.34–1.53)	0.396	1.06 (0.15–7.76)	0.954
9–14 days/month	1.04 (0.53–2.05)	0.906	2.92 (0.66–12.90)	0.157
≥15 days/month	0.98 (0.43–2.24)	0.967	0.74 (0.11–5.08)	0.763
Aura	1.78 (1.04–3.07)	0.036	5.80 (1.57–21.47)	0.009
Age (years)	0.99 (0.95–1.02)	0.479	1.02 (0.95–1.09)	0.635
Female sex	0.94 (0.53–1.68)	0.830	6.22 (0.70–54.89)	0.100
Education level (years)	0.95 (0.86–1.06)	0.374	0.76 (0.62–0.94)	0.013
**MARRIAGE STATUS**				
Single	Reference	–	Reference	–
Partnered/married	1.70 (0.83–3.48)	0.146	3.03 (0.56–16.41)	0.197
Divorced/widowed	3.62 (0.96–13.68)	0.058	3.94 (0.34–45.43)	0.272
**EMPLOY STATUS**				
Unemployed	Reference	–	Reference	–
Employed	0.53 (0.18–1.55)	0.248	1.42 (0.20–10.14)	0.728
Retired/homemaker	0.28 (0.06–1.19)	0.084	0.14 (0.01–2.92)	0.204
**ALCOHOL CONSUMPTION**				
Never	Reference	–	Reference	–
<Once a month	0.80 (0.45–1.43)	0.453	0.35 (0.07–1.73)	0.199
≥1 day a week	0.48 (0.11–2.21)	0.346	0.78 (0.06–10.60)	0.854
MIDAS	1.00 (0.99–1.01)	0.975	0.99 (0.98–1.01)	0.541
BDI total score	1.17 (1.10–1.23)	<0.001	1.11 (1.00–1.24)	0.042
HADS-anxiety	1.03 (0.94–1.13)	0.539	0.92 (0.74–1.15)	0.463
HADS-depression	1.04 (0.95–1.14)	0.414	0.99 (0.82–1.20)	0.931
PSQI total score	0.96 (0.88–1.04)	0.286	1.12 (0.91–1.39)	0.291

*OR, odds ratio; CI, confidence interval; MIDAS, Migraine Disability Assessment questionnaire; HADS, Hospital Anxiety and Depression Subscale; BDI, Beck Depression Inventory; PSQI, Pittsburgh Sleep Quality Index*.

## Discussion

### Main Research Findings

This study showed that migraine aura and depression severity (as indicated by the BDI total score) significantly predicted suicidal ideation in patients with migraine, after adjustment for possible confounding factors, such as age, sex, marital status, education level, employment status, alcohol consumption, BDI scores, HADS-anxiety and -depression subscores, and PSQI total scores. Patients with migraine who said they had attempted suicide were more likely to have migraine aura, low education level, and a high BDI total score. We show that there was an association between migraine patients with aura and suicidal ideation and suicide attempt. Our study also found an association between migraine patients with depression and suicidal ideation and suicide attempt, similar to a previous study conducted in the Peruvian population (27). In contrast, there was no association between migraine (with and without aura) occurring with high frequency and suicidal ideation and suicide attempt.

Notably, this study observed the significance of an aura in the following ways: (1) Migraine frequency was related to suicidal ideation and suicide attempts only in patients who experienced an aura. The highest prevalence of suicidal ideation (47.2%) and attempted suicide (13.9%) was observed in chronic migraine patients who had auras. (2) The presence of an aura itself was an independent predictor of suicidal ideation and suicide attempts. (3) In contrast, patients who had attempted suicide were more likely to experience an aura than patients who had not. (4) In patients who did not experience an aura, the frequency of migraines did not predict suicidal ideation or suicide attempts, after other factors were accounted for.

In our study, the adjusted odds ratio (OR) for suicide attempts as a function of experiencing an aura was 5.80 (*p* = 0.009; 95% confidence interval: 1.57–21.47). Overall, this finding is in contrast to a recent longitudinal study (7) which found no difference in suicide risk in patients who had an aura. There are several possible reasons for the discrepancy. First, our study recruited younger adults with migraine (mean age: 33.8 vs. 40.4 years). Second, the present study was clinic-based, and the patients were therefore, more likely to have severe symptoms. Third, Asian and Western populations have genetic and sociocultural differences.

Migraine headache has many comorbidities, the most common being depression and anxiety; 63.8 % of migraine patients have depression and 60.4% have anxiety (28). Studies have found that migraine headache is linked to suicide risk and psychiatric comorbidities, including depression, anxiety, and sleep quality (6, 7, 9, 16, 29). We observed similar results in our univariate analyses. Previous studies found that high scores for MIDAS, HADS, BDI, and PSQI scores were associated with high migraine frequency in adults (6, 30). Similarly, migraine is linked to psychiatric comorbidities in children and adolescents. A previous review revealed that migraine is associated with psychiatric disorders, including depression, anxiety disorders, periodic limb movement disorder, attention deficit hyperactivity disorder, and Tourette syndrome in children and adolescents (31). Wang et al. found that 13–15 years-old migraine patients with auras and high headache frequencies (>7 days/month) were more likely to exhibit suicidal ideation than were those without these features (16). Breslau et al. found that in 21–30 years-old migraine patients, the presence of an aura was significantly associated with suicide attempts (29).

Our study found that patients with migraines of all types had much higher levels of suicidal ideation than healthy controls. A hospital-based study in Korea also found that migraine patients had greater suicidal ideation (17.3%) than a healthy control group (3.8%; OR = 5.09, *p* = 0.003) (32). Furthermore, our study found that patients with migraine and a low education level had a higher risk of attempting suicide than those with higher education levels (adjusted OR = 0.76, *p* = 0.013). Kim et al. also found a high prevalence of suicidal ideation among migraine patients with a lower level of education (OR = 0.39, *p* = 0.029) (32).

### Putative Pathophysiological Mechanisms

Migraine and suicide may share some genetic and neurochemical pathophysiological mechanisms. First, abnormalities of the central serotonergic system have been implicated in both. Low levels of 5-hydroxyindolacetic acid in the cerebrospinal fluid have been associated with suicide (33). Low activity in the serotonergic descending pain inhibitory system is thought to play a role in migraine pathophysiology (34). In addition, patients with migraine have low plasma serotonin between migraine attacks and high serotonin concentration during attacks; hence, selective serotonin agonists (triptans) are used to treat migraine (35, 36). A study found that the plasma serotonin levels in patients with migraine with aura were significantly lower than in patients with migraine without aura or in a control group (37). Two studies have reported that the distribution of polymorphism frequencies in the serotonin transporter-linked promoter region is significantly different between patients who do and do not experience an aura (38, 39). Collectively, these findings corroborate the fact that low serotonin levels are associated with migraine attacks, particularly in patients with auras. Second, dysfunction of the hypothalamic–pituitary–adrenal axis may be associated with both migraine and suicide (40–42). Third, pain severity and frequency may be correlated with suicidality. A population-based study found a strong relationship between the chronic pain of migraine and suicidal ideation and suicide attempts, after adjustment for mental disorders (43). A community-based study showed a relationship between migraine aura and high suicidal risk in adolescents with chronic daily headaches (44). Consequently, the literature generally supports our finding that migraine frequency in patients with auras is associated with suicidal ideation and suicide attempts. Fourth, dopaminergic polymorphisms may be implicated in both migraine and suicide. A biologically based study showed that a specific dopamine D2 receptor genotype (DRD2 NcoI allele) was associated with comorbid migraine with aura, major depression, and generalized anxiety disorder (45).

### Strengths and Limitations

The present study has some strengths, namely, a well-controlled study design; demographic homogeneity; aura-based and frequency differentiated subgroup analyses; large cohort size; validated questionnaires; consideration of comorbid depression, anxiety, and sleep quality; and robust statistical analysis. However, it also has several limitations. First, the cross-sectional design limits the ability to establish a causal relationship between migraine and suicide risk. Hence, a longitudinal study is required. Second, this was a hospital-based study, and thus the results may not be representative of the entire migraine population. Thus, further population-based research is warranted. Third, suicidal ideation and suicide attempts were evaluated using questionnaires rather than through psychiatric standardized evaluation. Therefore, the patients may not have provided consistently accurate information. However, this approach has been used in previous studies (25, 26, 46, 47). Further studies employing psychiatric standardized evaluation are warranted to clarify suicide risk among patietns with migraine. Fourth, chronic pain disorders other than migraine, substance use disorders other than alcohol use disorder, bipolar disorder, and prophylactic medications use for migraine (e.g., antiepileptic drugs) were not controlled for in the multivariable analysis. Additional studies are warranted to control these possible confounding factors. Finally, the chronic migraine group, particularly the subgroup with auras, included a relatively small number of participants. Future studies with larger sample sizes of patients with chronic migraine and with auras are warranted.

### Conclusion

In conclusion, in migraine patients with auras, higher migraine frequency is associated with greater suicidality, including suicidal ideation and suicide attempts. High BDI total score and migraine aura are independent predictors of suicidal ideation and suicide attempts in patients with migraines. The results of this study imply the importance of evaluating the suicidality in patients with migraine auras, particularly in high migraine frequency patients who visit an outpatient clinic.

## Data Availability

All datasets generated for this study are included in the manuscript and/or the supplementary files.

## Author Contributions

Y-KL and F-CY: conception/design; F-CY: provision of study materials; F-CY: the guarantor of the paper, taking responsibility for the integrity of the work as a whole, from inception to published paper; All authors: collection and/or assembly of data, data analysis and interpretation, manuscript preparation, and final approval of manuscript. All authors have contributed substantially to, and are in agreement with the content of, the manuscript.

### Conflict of Interest Statement

The authors declare that the research was conducted in the absence of any commercial or financial relationships that could be construed as a potential conflict of interest.
