# Supplementary material for: Association of Suicide Risk With Headache Frequency Among Migraine Patients With and Without Aura
Source: Front Neurol. 2019 Mar 19;10:228. doi: 10.3389/fneur.2019.00228 (PMC6433743; doi:10.3389/fneur.2019.00228)
Supplement: Supplementary file 1 [file Table_1.DOCX]

**Supplemental Table 1**. Prevalence of suicidal ideation and suicide attempts in the male subjects according to the control and migraine groups

|  |  | Episodic migraine | | |  |  |  |
| --- | --- | --- | --- | --- | --- | --- | --- |
| Outcome / subgroup | Control | 1–4 days/month | 5–8 days/month | 9–14 days/month | ≥ 15 days /month | *P* value§ | *P* value‡ |
| Suicidal ideation |  |  |  |  |  |  |  |
| Total | 3 (3.4) | 18 (24.3) | 6 (14.6) | 4 (11.4) | 7 (23.3) | 0.039 | 0.908 |
| With aura | 3 (3.4) | 8 (33.3) | 2 (15.4) | 2 (20.0) | 5 (41.7) | <0.001 | 0.988 |
| Without aura | 3 (3.4) | 10 (20.0) | 4 (14.3) | 2 (8.0) | 2 (11.1) | 0.256 | 0.885 |
| Suicide attempt |  |  |  |  |  |  |  |
| Total | 0 (0.0) | 1 (1.4) | 0 (0.0) | 0 (0.0) | 1 (3.3) | 0.259 | 0.996 |
| With aura | 0 (0.0) | 0 (0.0) | 0 (0.0) | 0 (0.0) | 0 (0.0) | NA | NA |
| Without aura | 0 (0.0) | 1 (2.0) | 0 (0.0) | 0 (0.0) | 1 (5.6) | 0.169 | 0.999 |

§ Linear trend of Cochran–Armitage chi-square test; ‡ Linear contrast in the logistic regression adjusted for age, marital status, years of education, employment status, alcohol consumption, Hospital Anxiety and Depression Subscales; Beck Depression Inventory scores, Hospital Anxiety and Depression Subscales for anxiety and depression, and Pittsburgh Sleep Quality Index total scores; NA, not applicable.

**Supplemental Table 2**. Prevalence of suicidal ideation and suicide attempts in the female subjects according to the control and migraine groups

|  |  | Episodic migraine | | |  |  |  |
| --- | --- | --- | --- | --- | --- | --- | --- |
| Outcome / subgroup | Control | 1–4 days/month | 5–8 days/month | 9–14 days/month | ≥ 15 days /month | *P* value§ | *P* value‡ |
| Suicidal ideation |  |  |  |  |  |  |  |
| Total | 2 (2.2) | 19 (14.6) | 10 (15.4) | 25 (28.7) | 20 (30.3) | <0.001 | 0.046 |
| With aura | 2 (2.2) | 6 (15.4) | 7 (30.4) | 11 (44.0) | 12 (50.0) | <0.001 | 0.027 |
| Without aura | 2 (2.2) | 13 (14.3) | 3 (7.1) | 14 (22.6) | 8 (19.0) | 0.001 | 0.251 |
| Suicide attempt |  |  |  |  |  |  |  |
| Total | 0 (0.0) | 3 (2.3) | 2 (3.1) | 7 (8.0) | 5 (7.6) | 0.002 | 0.117 |
| With aura | 0 (0.0) | 2 (5.1) | 2 (8.7) | 4 (16.0) | 5 (20.8) | <0.001 | 0.015 |
| Without aura | 0 (0.0) | 1 (1.1) | 0 (0.0) | 3 (4.8) | 0 (0.0) | 0.205 | 0.871 |

§ Linear trend of Cochran–Armitage chi-square test; ‡ Linear contrast in the logistic regression adjusted for age, marital status, years of education, employment status, alcohol consumption, Hospital Anxiety and Depression Subscales; Beck Depression Inventory scores, Hospital Anxiety and Depression Subscales for anxiety and depression, and Pittsburgh Sleep Quality Index total scores.
